# Comparative Efficacy of Different Pharmacological Treatments for Pityriasis Rosea: A Network Meta-Analysis

**DOI:** 10.3390/jcm13226666

**Published:** 2024-11-06

**Authors:** Giulia Ciccarese, Antonio Facciorusso, Astrid Herzum, Cristian Fidanzi, Sebastiano Recalcati, Caterina Foti, Francesco Drago

**Affiliations:** 1 Section of Dermatology, Department of Medical and Surgical Sciences, University of Foggia, Viale Pinto 1, 71122 Foggia, Italy; 2 Liver Unit, C.U.R.E. (University Centre for Liver Disease Research and Treatment), Department of Medical and Surgical Sciences, University of Foggia, 71122 Foggia, Italy; antonio.facciorusso@unifg.it; 3 Dermatology Unit, IRCCS Giannina Gaslini, 16100 Genoa, Italy; astridherzum@yahoo.it; 4 Unit of Dermatology, Hospital of Carrara, 54033 Carrara, Italy; cri.fidanzi@outlook.it; 5 Unit of Dermatology, Alessandro Manzoni Hospital, ASST Lecco, 23900 Lecco, Italy; sebastianorecalcati@gmail.com; 6 Section of Dermatology and Venereology, Department of Precision and Regenerative Medicine and Ionian Area (DiMePRe-J), University of Bari “Aldo Moro”, 70121 Bari, Italy; caterina.foti@uniba.it; 7 Casa di Cura Villa Montallegro, 16100 Genoa, Italy; francescodrago007@gmail.com

**Keywords:** pityriasis rosea, acyclovir, treatment

## Abstract

**Background/Objectives**: Pityriasis rosea (PR) is a self-limiting exanthematous disease associated with the endogenous reactivation of human herpesviruses (HHV)-6 and HHV-7. Classically, the lesions gradually resolve, leaving no sequelae. Therefore, the best treatment is reassuring the patient and suggesting a resting period. However, atypical PR cases characterized by extensive, persistent lesions and systemic symptoms may impact the patient’s quality of life, and, therefore, a treatment can be prescribed. There is limited evidence on the comparative effectiveness of pharmacological treatments for PR; therefore, we performed a network meta-analysis to compare these interventions. **Methods**: Overall, 12 randomized control trials (RCTs) were identified. The outcomes were itch resolution and rash improvement. Results were expressed as risk ratio (RR) and 95% confidence interval (CI). We also calculated the relative ranking of the interventions for achieving the aforementioned outcomes as their surface under the cumulative ranking (SUCRA). **Results**: On network meta-analysis, only oral steroids and the combination of oral steroids+antihistamine resulted significantly superior to the placebo in terms of itch resolution (RR 0.44, CI 0.27–0.72 and RR 0.47, CI 0.22–0.99). Oral steroids resulted in the best treatment (SUCRA 0.90) for itch resolution. In terms of rash improvement, only acyclovir and erythromycin resulted significantly superior to placebo (RR 2.55, CI 1.81–3.58; and RR 1.69, CI 1.23–2.33), and acyclovir outperformed all the other tested interventions. Consequently, acyclovir ranked as the best intervention (SUCRA score 0.92). **Conclusions**: Acyclovir represents the best option for patients with PR that have extensive, persistent lesions or systemic symptoms. Steroids and antihistamines seemed the best treatment for itch resolution.

## 1. Introduction

Pityriasis rosea (PR) is an acute, self-limiting exanthematous disease associated with the endogenous systemic reactivation of human herpesvirus (HHV)-6 and/or HHV-7 [1,2]. The causal role of the systemic active HHV-6 and HHV-7 infections in the pathogenesis of PR is supported by a large body of evidence using the most modern biological techniques [1,2,3,4]. Among these results, the cytopathic effects revealed in culture of peripheral blood mononuclear cells, the detection of HHV-6 and HHV-7 DNA in plasma of patients with PR, and the presence of their viral antigens and mRNA expression in PR skin lesions are all markers of active viral replication [1,2,3,4]. In addition, the upregulation of the serum levels of interleukin (IL)-17, IL-22, IL-36, IFN-*γ*, vascular endothelial growth factor, CX3CL1/fractalkine, and CXCL10 in patients with PR compared to the controls is further evidence that PR is associated with the activation of cellular immunity and induction of an inflammatory response against a virus [5,6].

**Figure 1:**
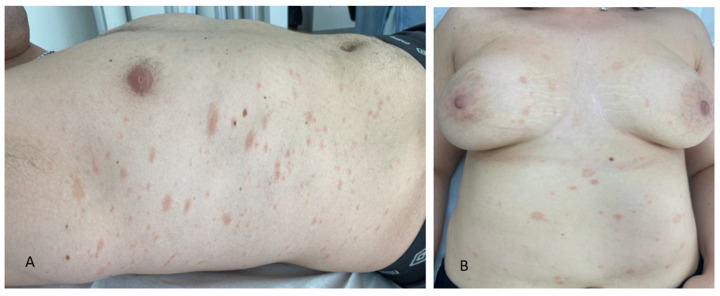
Erythematous macular and papular scaly lesions of the trunk with a ‘theatre curtain’ distribution in a young man (**A**) and in woman (**B**) with PR.

The cutaneous eruption of PR usually begins with a solitary erythematous papule (‘herald patch’ or ‘mother patch’) that enlarges rapidly to form an annular or oval lesion measuring between 2 and 10 cm in diameter with an erythematous, salmon-colored, scaling borders, and a paler, slightly depressed center. This primary lesion remains isolated for about 2 weeks, followed by a generalized secondary eruption consisting of smaller, scaly papulosquamous lesions oriented with their long axis along the Langer’s lines of cleavage of the trunk in a ‘theatre curtain’ pattern [1]. The eruption is most often limited to the trunk, neck, and proximal area of the limbs, usually sparing the face (Figure 1).

Maculo-papular and petechial oropharyngeal lesions are observed in 28% of patients [7]. Prodromal symptoms such as general malaise, fatigue, headache, difficulty concentrating, gastrointestinal and upper respiratory symptoms, and mild fever may precede the exanthema. The exanthem is sometimes associated with mild pruritus [1,7]; however, pruritus may be more severe, especially if the skin lesions have been treated with a topical medication, such as antimycotic creams. The typical eruption lasts about 45 days, but a short duration of 2 weeks and persistent eruptions of several months have been described [1]. PR affects mainly young adults, and the diagnosis is entirely clinical [1]. Many forms considered atypical for morphology, size, number, distribution, symptom severity, and course exist [8,9]. However, it is necessary to distinguish from these the PR-like eruptions, which can clinically resemble typical PR but which have a completely different pathogenesis, course, and prognosis, being a drug-induced or a vaccine-induced rash; criteria to distinguish between PR and PR-like eruptions have been described taking into account clinical, histopathologic, and virologic features [10].

The typical PR is a self-limited and frequently asymptomatic disease, and the benefits associated with the use of any active intervention should therefore carefully consider any potential adverse effects and the cost of the drug. The best treatment is, therefore, reassuring the patient regarding the nature of the condition and recommending some rest [1,10]. However, in particular cases characterized by extensive and persistent lesions and in those associated with systemic symptoms, the disease may have a significant impact on the patient’s quality of life, and, therefore, a treatment can be prescribed [1,10].

Noteworthy, PR has been reported to occur more frequently in pregnancy than in the general population (18% versus 6%) [11], and when it occurs in pregnant women, it may justify some concern. Indeed, since pregnancy is a state of an altered immune response, the risk of HHV-6/7 persistent reactivation can exist, and intrauterine transmission of HHV-6 and -7 after viral reactivation in the mother has been reported on several occasions [12,13,14,15]. In fact, it has been shown that 14% of HHV-6 congenital infection results from intrauterine infections from the mother [16]. In women developing PR during pregnancy, the most important risk factors threatening the successful outcome of pregnancy are just the high viral load of HHV-6 in the plasma [12]. The onset of PR before week 15 and the presence of oropharyngeal lesions are additional major risk factors that must be taken into account [12]. Systemic symptoms, extensive widespread of the lesions, and PR long duration are statistically lower risk for unfavorable pregnancy outcome [12,17,18]. To date, there are no specific guidelines for the treatment of PR during pregnancy, but there is some evidence for the benefit of acyclovir, a drug considered safe in pregnancy [12,19,20].

Several studies evaluated the efficacy of topical and systemic treatment for PR and found conflicting results [21,22,23,24,25,26,27,28,29,30,31,32,33]. To date, a network meta-analysis that compares at the same time the effectiveness of several treatments for PR has never been performed. Therefore, we decided to realize a pairwise and network meta-analysis comparing the effectiveness of several pharmacological treatments of PR in terms of improvement of skin eruption (reduction in number and size of the lesions and reduction of erythema and desquamation) and itch resolution within two weeks from the diagnosis. In contrast to pairwise meta-analyses, network meta-analysis can inform the simultaneous comparative performance of multiple interventions and synthesize evidence across a network of randomized control trials (RCTs), also providing a ranking of the effectiveness of the tested interventions.

The quality of evidence of our findings was also assessed, and Grading of Recommendations Assessment, Development, and Evaluation (GRADE) criteria for network meta-analysis were used to appraise the quality of evidence [34].

## 2. Materials and Methods

This systematic review was reported according to the Preferred Reporting Items for Systematic Reviews and Meta-Analyses (PRISMA) statement [35].

Inclusion and exclusion criteria

Our focused question on the comparative effectiveness of different pharmacological treatments for PR was addressed following the Population, Intervention, Comparator, and Outcome (PICO) format. Included studies were parallel RCTs published as full-text papers or conference abstracts that met the following inclusion criteria: (A) Patients: adult/adolescent/child patients with PR that underwent an intervention; (B) Interventions: acyclovir, antihistamine, azithromycin, clarithromycin, erythromycin, steroids, steroids + antihistamine; (C) Comparators: placebo or compared to each other; (D) Outcomes: itch resolution and rash improvement.

We excluded observational non-randomized studies, articles in which neither abstract nor text was written in the English language, and studies that did not report the outcomes at 2 weeks.

Search Strategy

A computerized bibliographic search was performed on PubMed/Medline, Scopus, and Web of Science from inception to July 2024, using the search string reported in the Supplementary Materials (Supplementary Table S1).

Two investigators (GC, and FD) independently selected articles of interest. In cases of multiple publications from the same authors, only the most recent and complete article was included.

Data Abstraction and Risk of Bias Assessment

Data on study-, participant-, and intervention-related characteristics were abstracted onto a standardized form by two investigators (GC and AF) independently; discrepancies were resolved by consensus, referring back to the original article, in consultation with a third reviewer (FD). The quality of the included studies was assessed by two authors independently (GC and FD) according to the Cochrane Collaboration’s tool 2 for assessing the risk of bias [36].

Outcomes

Primary outcomes of interest were itch resolution within two weeks, defined as the absence of cutaneous symptoms as rated by the patient (for example through the visual analogue scale [VAS]), and improvement of the eruption within two weeks, defined as a reduction in number and size of the lesions and reduction of erythema and desquamation.

The choice of 2 weeks as the timing of the outcome assessment is due to the consideration that, although the typical PR lasts about 45 days, a shorter duration of 2 weeks has been described [1,8,10,12]; moreover, without any active treatment, patients with PR usually start having spontaneous recovery between 2 and 12 weeks [1,33]. Therefore, any improvement after two weeks of active treatment would make it difficult to differentiate whether the improvement is due to spontaneous recovery from the disease or to the treatment. Furthermore, not all the patients were diagnosed with PR at the onset of the disease; therefore, evaluating the outcome of a treatment more than two weeks after diagnosis would mean evaluating the patient after an indefinite number of days of spontaneous recovery.

Statistical Analysis

Pooled estimates were reported as relative risk (RR) and 95% CI, using the DerSimonian and Laird random-effects approach [37]. We assessed statistical heterogeneity using the I2 statistic, with values over 50% indicating substantial heterogeneity. Small study effects were assessed by examining funnel plot asymmetry.

We then conducted a network meta-analysis for itch resolution and improvement of the skin eruption through a frequentist approach based on a random-effects consistency model [38]. Network consistency was evaluated by comparing the direct estimates to the indirect estimates for each comparison, using a node-splitting technique. Network meta-analysis was performed with R 6.2-0 package netmeta (Foundation for Statistical Computing, Vienna, Austria).

We calculated the relative ranking of the interventions for achieving the aforementioned outcomes as their surface under the cumulative ranking (SUCRA). SUCRA values range between 0 when a treatment is certainly the worst and 1 when a treatment is certainly the best [38].

Safety data were inconsistently reported and only descriptively analyzed in the Supplementary Materials.

Quality of Evidence

The quality of evidence for itch resolution and rash improvement derived from pairwise and network meta-analysis was judged using the GRADE framework [39] (Supplementary Table S2). Evidence from RCTs started at high quality, and it was rated down for the presence of any of the following aspects: risk of bias in the literature, inconsistency, indirectness, inaccuracy, and bias in publication [39].

## 3. Results

### 3.1. Characteristics of Included Studies

**Figure 2:**
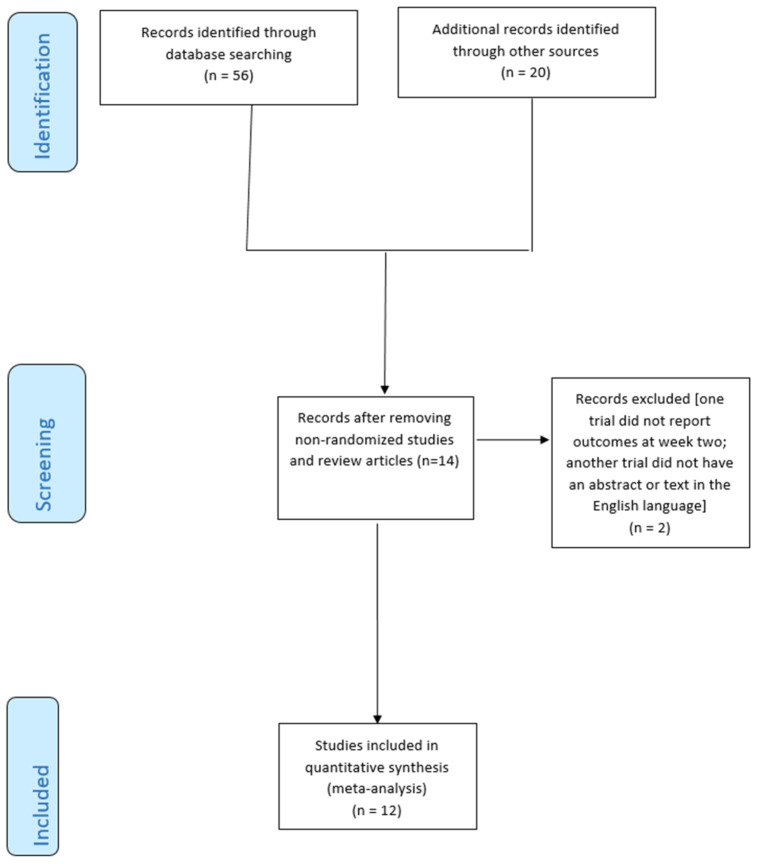
Flow chart of included studies.

From 76 studies identified using our search strategy, 40 records were excluded based on titles and abstracts, and 6 because they were duplications; a further 16 studies were excluded because there was no mention of randomization. The research identified 14 studies; however, we excluded one trial that did not report outcomes at week two [40] and another trial that did not have an abstract or text in the English language [41]. Finally, 12 RCTs [21,22,23,24,25,26,27,28,29,30,31,32] (638 patients) comparing seven different treatments and placebo were included for quantitative synthesis (Figure 2).

**Figure 3:**
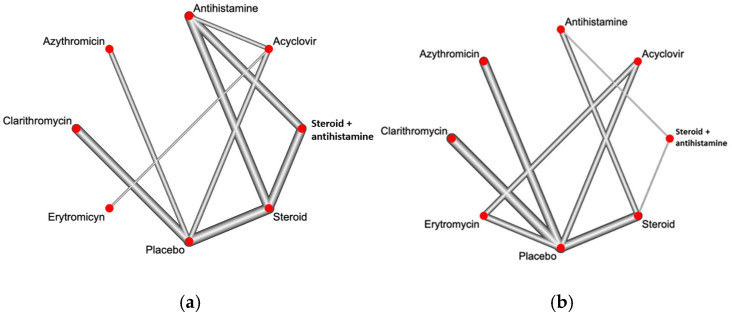
Network of included studies with comparisons between pharmacological treatments in inducing itch resolution (**a**) and rash improvement (**b**). The size of the nodes and the thickness of the edges are weighted based on the number of studies evaluating each intervention and direct comparison, respectively.

Figure 3a,b shows the available direct comparisons and network of trials for itch resolution and improvement of the eruption, respectively.

The main characteristics of included RCTs are reported in Table 1.

The recruitment period ranged from March 1993 to September 2013. The two arms in the parallel RCTs were equable in terms of baseline aspects (mean age and sex). Five RCTs used the instrument of the VAS [22,29,30,31,32], while four RCTs did not specify the methods of pruritus assessment [21,24,25,27]; three RCTs did not evaluate the outcome of itch [23,26,28]. To evaluate the improvement of the skin eruption, only three RCTs used a standardized scale as the pityriasis rosea area and severity index (PRASI) [31] or the pityriasis rosea severity index (PRSS) [29,32]; most of the RCTs assessed the rash improvement by counting the lesions at the onset of disease and at the control visit, also using digital photographs [21,22,23,24,25,30]; a decrease in erythema, size, and scaling of the lesions were sometimes considered for assessing the achievement of the outcome [26,28].

As reported in Supplementary Table S3, a risk of bias assessment was performed in the context of the primary outcomes, and overall, studies were felt to be at low risk of bias. Even if unblinded, none of the RCTs showed deviations from the intended protocol.

### 3.2. Itch Resolution

As reported in Table 2, when combining direct and indirect evidence through network meta-analysis, only oral steroids and the combination of oral steroids + antihistamine resulted significantly superior to placebo (RR placebo vs. steroids 0.44, CI 0.27–0.72 and RR placebo vs. steroids + antihistamine 0.47, CI 0.22–0.99), whereas none of the other interventions significantly outperformed placebo (Table 2).

Among the other direct and indirect comparisons concerning itch resolution, antihistamines resulted significantly superior to erythromycin (RR 17.96, CI 1.07–299.68), clarithromycin and erythromycin was found to be inferior to steroids (RR 0.37, CI 0.17–0.81 and RR 0.03, CI 0.01–0.58, respectively); furthermore, erythromycin was significantly inferior to the combination of oral steroids + antihistamines (RR 0.03, CI 0.01–0.64; Table 2).

Consequently, as reported in Table 3, oral steroids resulted as the best option for itch resolution (SUCRA 0.90), followed by oral steroids+ antihistamines (SUCRA 0.84) and antihistamines (SUCRA 0.67). Among the other treatments, only acyclovir ranked better than placebo (SUCRA 0.45 vs. 0.40), whereas erythromycin showed the poorest performance in terms of itch resolution (SUCRA score 0.02).

### 3.3. Improvement of the Skin Eruption

As reported in Table 4, only acyclovir and erythromycin resulted significantly superior to placebo (RR 2.55, CI 1.81–3.58 and RR 1.69, CI 1.23–2.33, respectively).

Acyclovir outperformed all the other tested interventions (RRs ranging from 1.50 to 19.39 and CIs always beyond 1). The combination of oral steroids and antihistamines was statistically inferior to all the other treatments except clarithromycin (RR clarithromycin vs. steroids+antihistamines 6.72, CI 0.95–47.2).

Among the other comparisons, clarithromycin was significantly inferior to erythromycin (RR 0.52, CI 0.34–0.77) and oral steroids (RR 0.54, CI 0.37–0.78).

Consequently, as reported in Table 3, acyclovir ranked as the best intervention (SUCRA score 0.92), followed by erythromycin (SUCRA 0.79) and steroids (SUCRA 0.73). Clarithromycin (SUCRA 0.30) and oral steroids + antihistamines (SUCRA 0.01) were inferior to placebo (SUCRA 0.38).

### 3.4. Small Study Effects, Network Coherence, Safety Outcomes, and Quality of Evidence

We found no evidence of small study effects for the primary outcomes through inspection of funnel plots. Direct and indirect estimates did not show a significant difference, and where applicable, there was no intransitivity observed between the findings of direct and indirect meta-analyses (*p* = 0.31).

All tested medications were deemed safe, with only a few mild adverse events (AEs), primarily gastrointestinal, as shown in Supplementary Table S4. Importantly, these AEs did not necessitate discontinuation of the drugs.

The quality of evidence was downgraded for imprecision related to broad CI crossing unity or failure to meet the optimal information size, as well as for indirectness related to heterogeneous definitions of outcomes in the included RCTs. No inconsistency, risk of bias in the literature, or publication bias was identified. Consequently, the overall body of evidence was deemed to be of low quality.

## 4. Discussion

Though self-limiting in about 6–8 weeks, PR may have a prolonged clinical course, recurrences may occur (usually within one year), and the extension of the lesions may be generalized and associated with systemic symptoms with a significant impact on the patients’ quality of life [1,8,9,10]. Moreover, in pregnant women, PR may be associated with pregnancy complications, such as fetal distress, oligohydramnios, and premature delivery [17,28], and in some cases fetal deaths, abortions, or miscarriages [11,12]. In all these atypical courses of PR, an effective treatment could be considered.

The most recent Cochrane Review on the interventions for PR included 14 trials (761 participants) and assessed the efficacy of macrolide antibiotics, acyclovir, phototherapy, oral steroids, antihistamines, and Chinese medicine. The authors concluded that oral acyclovir leads to good or excellent rash improvement [31]. In such a study, the effectiveness of the different drugs has been compared in pairs (for example, azithromycin versus placebo, acyclovir plus antihistamine versus antihistamine, and others) [33]. Conversely, the present work is the first network meta-analysis that simultaneously compares the performance of multiple pharmacological treatments for PR and summarizes evidence across a network of RCTs, providing a ranking of the effectiveness of the treatments.

Using network meta-analysis with GRADE methodology to optimally inform and critically appraise evidence, we made several key observations.

Firstly, in terms of itch resolution, only oral steroids (alone or in combination with antihistamines, for example oral betamethasone/prednisolone or oral betamethasone + dexchlorpheniramine) resulted significantly superior to placebo (RR placebo vs. steroids 0.44, CI 0.27–0.72 and RR placebo vs. steroids + antihistamines 0.47, CI 0.22–0.99), whereas none of the other interventions significantly outperformed placebo. Antihistamines alone (oral dexchlorpheniramine) were also effective and, although not significantly superior to placebo, they outperformed erythromycin; however, the large CI poses a note of caution in the interpretation of this finding. Oral steroids were also superior to macrolides. Overall, oral steroids alone resulted as the best intervention for itch resolution (SUCRA 0.90), followed by steroids+antihistamines (SUCRA 0.84) and antihistamines (SUCRA 0.67); among the other treatments, only acyclovir ranked better than placebo in improving the itch. Macrolides showed a very poor performance in terms of itch resolution.

Of note, corticosteroids are not directly antipruritic, and it is believed they exert a beneficial effect on pruritus through their reduction in skin inflammation [42]. However, because of their immunosuppressive effects, the administration of oral steroids could be inappropriate in a disease characterized by viral reactivations, like PR. Furthermore, HHV-6 and HHV-7 reactivations may be associated, especially in immunosuppressed patients (transplant recipients, patients undergoing oncological treatments), with severe complications like hepatitis, pneumonitis, and encephalitis, and, therefore, these viral reactivations can benefit from antiviral drugs rather than oral steroids [43,44].

Secondly, acyclovir showed striking results concerning rash improvement with a clear superiority over all the other tested interventions. Out of the other drugs, only erythromycin was significantly superior to the placebo (RR 1.69, CI 1.23–2.33). The very poor performance of the combination of steroids and antihistamines deserves some explanation. First of all, it is based on a single small study, and this result needs further validation; secondly, the immunosuppressive effect of steroids may exacerbate (instead of recover) a disease like PR associated with endogenous viral reactivations and therefore prolong its course [10].

The present results strongly corroborate our previous studies. In 2006, Drago et al. [45] evaluated 87 consecutive patients with PR who were treated for 1 week with oral acyclovir (800 mg five times daily) or placebo (vitamin C tablets); on the 7th day of observation, there were significantly fewer new lesions in patients treated in the first week from onset than in patients treated afterwards. Remarkably, on the 14th day of therapy, 79% of treated patients completely regressed compared to 4% of the patients who took placebo. Although this study was not a randomized, double-blind controlled trial (objectivity was accomplished through counting the skin lesions), it first revealed the efficacy of acyclovir in the treatment of PR, especially in patients treated during the first week from onset when replicative activity of HHVs is elevated [45]. In the following years, RCTs, systematic reviews, and meta-analyses further confirm the data on the efficacy of oral acyclovir during the early course of PR [30,46,47]. Among the antivirals available against human herpesviruses, ganciclovir, foscarnet, and cidofovir proved to be active in inhibiting HHV-6 replication both in laboratory-based studies and in studies carried out in living organisms. However, the use of these agents, though more active than acyclovir, can be characterized by severe side effects such as myelosuppression and nephrotoxicity [45]. Conversely, acyclovir at high doses has an anti-HHV-6 effect inhibiting viral DNA synthesis and viral replication; it also exhibits easy availability and a low rate of side effects [45,48,49]. Acyclovir (9-[{2-hydroxyethoxy}methyl]-9H-guanine) is one of the most commonly used anti-herpetic nucleoside analogs in clinical practice. It is the prototype of a group of antiviral agents that are activated by viral thymidine kinase to become inhibitors of viral DNA polymerase that block viral DNA synthesis [50].

A lower activity of acyclovir against HHV-7 has also been shown [48,49,50]. However, it has been demonstrated that HHV-7 replication may precede and stimulate HHV-6 reactivation; once reactivated, HHV-6 genomes may predominate and replace the former, leading HHV-7 to disappear or impair its detection by PCR [1,13]. Therefore, the use of an antiviral agent like acyclovir, which is mainly directed against HHV-6, is justified.

The comparison between different macrolides showed a superiority of erythromycin over clarithromycin, which was also found to be inferior to steroids (RR 0.54, 0.37–0.78). Consequently, acyclovir ranked as the best intervention (SUCRA score 0.92) in rash improvement, followed by erythromycin (SUCRA 0.79) and steroids (SUCRA 0.73). Clarithromycin (SUCRA 0.30) and steroids + antihistamines (SUCRA 0.01) were the poorest treatments, even inferior to placebo. The efficacy shown by some macrolide antibiotics in PR could be explained by their modest anti-inflammatory and immunomodulatory properties through inhibition of IL-6 and IL-8 secretion by bronchial epithelial cells and reduction of neutrophil activity [29,51]. The antipruritic effects of macrolides have been investigated in several pruritic skin diseases (psoriasis vulgaris, atopic dermatitis, prurigo nodularis) showing contradictory results. The exact reason for the antipruritic effect of macrolides has not been elucidated; however, it could be related to its inhibitory action on the production of cytokines or neuropeptides that cause pruritus [51].

Finally, given the large amount of evidence on the PR viral etiology, the administration of antibiotics is not recommended.

Our study has some limitations, correlated to both the network meta-analysis and individual studies. First, there were a small number of head-to-head trials supporting some of the comparisons, and many of the included RCTs were underpowered. Second, most of the studies included were unblinded RCTs, so prone to performance bias. However, no deviations from the intended protocol were detected in any of the included RCTs, so the evidence was not downrated for this specific item. Third, the definition of the outcome “rash improvement” and the methods to evaluate the achievement of this outcome were heterogeneous across the various trials. Conversely, as regards the itch, the outcome was evaluated as “itch resolution” (not as “itch improvement”); therefore, the achievement could be evaluated objectively also without a scale.

## 5. Conclusions

In conclusion, based on this systematic review with network meta-analysis of different interventions for PR, in terms of rash improvement, acyclovir represents the best option to consider in cases of PR characterized by extensive skin eruption and systemic symptoms and in cases with recurrent or persistent course (800 mg 5 times daily for 7 days) [45]. Moreover, despite the absence of consensus on treatment, when PR develops during the first week’s gestation showing an aggressive course with generalized lesions and systemic symptoms, after consultation with the patient’s obstetrician, treatment with acyclovir may be cautiously considered. In addition to intervening on the course of PR, this can also reassure patients, particularly those anxious for the outcome of their pregnancy. Erythromycin and oral steroids, which could represent other options, are less effective than acyclovir. The use of clarithromycin should be abandoned due to its poor efficacy. Although oral steroids and antihistamines (alone or in combination) seemed the best treatment for itch resolution, acyclovir represents a valuable alternative (better than a placebo) that does not carry the risk of immunosuppressing the patients, leading to the possible systemic complications related to HHV-6/7 reactivation. Further large and adequately powered RCTs are needed to confirm these results.

## Figures and Tables

**Table 1:** Features of the included randomized control trials (RCTs).

Study, Year	Study Period, Country	Study Group	Sample Size	Mean Age, Years	Male Sex, *n* (%)	Inclusion Criteria	Exclusion Criteria	Definition of Itch Resolution	Definition of Improvement of the Eruption
Lazaro-Medina, 1996 [21]	March 1993–April 1995, the Philippines	oral dexchlorpheniramine	27	22	38 (45%)	classic PR ^1^	use of systemic/topic medication within the week before consultation; positive KOH preparation of skin scraping	not specified	change in lesion count
		oral betamethasone	31						
		oral betamethasone + dexchlorpheniramine	27						
Villarama, 2002 [22]	unknown total duration, the Philippines	oral erythromycin	20	24.6	18 (45%)	classic PR	atypical PR presentation; use of systemic/topic medication within 1 week before consultation; pregnant women	reduction in pruritus score (1 to 10 VAS ^2^)	decrease in size, erythema, size, scaling, and number of lesions, absence of new lesions
		placebo	20	27.1					
Akhyani, 2003 [23]	unknown total duration, Iran	oral erythromycin	23	11–36 (range)	22 (48%)	classic PR presenting within 1 week from onset	concern regarding other differential diagnosis (psoriasis, secondary syphilis, fungal infection)	itch was not evaluated	decrease in the number of lesions and erythema
		placebo	23						
Amer, 2006 [24]	unknown total duration, USA	oral azithromycin	25	8	16 (33%)	classic PR presenting within 3 weeks from onset	use of antibiotic within 2 weeks of PR diagnosis	not specified	decrease in the lesion number, scaliness or thickness
		placebo	24	8.4					
Ehsani, 2010 [25]	May 2007–April 2008, Iran	oral erythromycin	15	33	15 (50%)	PR within the first week from onset	pregnant women, concern regarding differential diagnosis	not specified	decrease in the lesion number
		oral acyclovir	15						
Rassai, 2011 [26]	October 2006–February 2007, Iran	oral acyclovir	28	27	unspecified	PR within 4 weeks from onset	use of systemic/topic medication; pregnant women	itch was not evaluated	decrease in erythema and scaling (lesions were digitally photographed)
		no intervention (follow-up)	26						
Ahmed, 2014 [27]	July 2008–July 2009, Pakistan	oral clarithromycin	30	23.3	33 (55%)	PR within 2 weeks from onset	pregnant women, use of systemic/topic medications within 2 weeks from diagnosis	not specified	decrease in the lesion number
		placebo	30	22					
Ganguly, 2014 [28]	November 2006–May 2008, India	oral acyclovir	38	unspecified	unspecified	PR	use of systemic medications in the preceding 2 weeks	itch was not evaluated	decrease in erythema
		placebo	35						
Pandhi, 2014 [29]	February 2010–March 2011, India	oral azithromycin	35	23	36 (51%)	PR	use of systemic medications in the preceding 2 weeks	reduction in pruritus score (1 to 10 VAS)	reduction in PR severity score (PRSS)
		placebo	35	23.6					
Das, 2015 [30]	March 2013–September 2013, India	oral acyclovir	12	32.5	14 (58%)	PR in patients > 18 years	pregnant women, concern regarding differential diagnosis	reduction in pruritus score (1 to 10 VAS)	decrease in the lesion number
		oral cetirizine	12	34					
Singh, 2016 [31]	August 2012–June 2013, India	oral acyclovir	14	24.4	19 (70%)	PR	pregnant women, use of systemic medications within 1 week from diagnosis; concomitance of other differential diagnosis (cutaneous fungal infection, syphilis)	reduction in pruritus score (0–3 scale)	decrease in the skin involvement, erythema, and scaling (pityriasis rosea area and severity index, PRASI)
		placebo	13	18.3					
Sonthalia, 2018 [32]	March 2011–March 2013, India	Oral prednisolone	35	26.03	34 (48%)	PR in patients > 18 years	pregnant women, concomitant of other differential diagnosis	reduction in pruritus score (1 to 10 VAS)	reduction in PR severity score (PRSS)
		placebo	35	25.8					

^1^ PR: pityriasis rosea; ^2^ VAS: visual analogue scale.

**Table 2:** Outcomes of network meta-analysis concerning itch resolution.

Itch Resolution							
Acyclovir	Antihistamine	Azithromycin	Clarithromycin	Erythromycin	Placebo	Steroid	Steroid + Antihistamine
Acyclovir							
0.72 (0.29–1.79)	Antihistamine						
1.22 (0.30–4.87)	1.69 (0.46–6.18)	Azithromycin					
1.21 (0.43–3.39)	1.67 (0.67–4.16)	0.98 (0.28–3.41)	Clarithromycin				
13 (0.90–186.42)	**17.96 (1.07–299.68)**	10.58 (0.52–212.23)	10.71 (0.61–186.08)	Erythromycin			
1.02 (0.44–2.37)	1.41 (0.7–2.83)	0.83 (0.28–2.47)	0.84 (0.46–1.52)	0.07 (0.01–1.28)	Placebo		
0.45 (0.18–1.11)	0.63 (0.36–1.1)	0.37 (0.11–1.23)	**0.37 (0.17–0.81)**	**0.03 (0.01–0.58)**	**0.44 (0.27–0.72)**	Steroid	
0.48 (0.17–1.33)	0.67 (0.36–1.25)	0.39 (0.10–1.48)	0.40 (0.15–1.03)	**0.03 (0.01–0.64)**	**0.47 (0.22–0.99)**	1.06 (0.59–1.89)	Steroid + Antihistamine

Results were expressed as risk ratio and 95% CI. Significant results were reported in bold. The numerator of the ratio was the column whereas the raw was the denominator.

**Table 3:** Ranking of interventions based on surface under the cumulative ranking (SUCRA) score.

Itch Resolution		Improvement of the Skin Eruption	
Steroid	0.9014	Acyclovir	0.9229
Steroids + antihistamine	0.8471	Erytromycin	0.7957
Antihistamines	0.6771	Steroids	0.7343
Acyclovir	0.4571	Antihistamine	0.4271
Placebo	0.4057	Azithromycin	0.4157
Azithromycin	0.3586	Placebo	0.3871
Clarithromycin	0.3314	Clarithromycin	0.3029
Erythromycin	0.0214	Steroid + antihistamine	0.0143

**Table 4:** Outcomes of network meta-analysis concerning rash improvement.

Rash Improvement							
Acyclovir	Antihistamine	Azithromycin	Clarithromycin	Erythromycin	Placebo	Steroid	Steroid + Antihistamine
Acyclovir							
**2.37 (1.25–4.50)**	Antihistamine						
**2.17 (1.39–3.38)**	0.91 (0.49–1.68)	Azithromycin					
**2.88 (1.89–4.38)**	1.21 (0.67–2.19)	1.32 (0.91–1.92)	Clarithromycin				
**1.50 (1.06–2.11)**	0.63 (0.33–1.18)	0.68 (0.45–1.05)	**0.52 (0.34–0.77)**	Erythromycin			
**2.55 (1.81–3.58)**	1.07 (0.62–1.84)	1.17 (0.88–1.55)	0.88 (0.69–1.12)	**1.69 (1.23–2.33)**	Placebo		
**1.57 (1.01–2.44)**	0.66 (0.41–1.05)	0.72 (0.48–1.07)	**0.54 (0.37–0.78)**	1.04 (0.68–1.59)	**0.61 (0.46–0.81)**	Steroid	
**19.39 (2.71–138.28)**	**8.16 (1.16–57.05)**	**8.91 (1.26–62.96)**	6.72 (0.95–47.2)	**12.92 (1.81–91.78)**	**7.6 (1.09–52.62)**	**12.31 (1.81–83.52)**	Steroid + Antihistamine

Results were expressed as risk ratio and 95% CI. Significant results were reported in bold. The numerator of the ratio was the column whereas the raw was the denominator.

## Data Availability

The original contributions presented in the study are included in the article/Supplementary Materials. Further inquiries can be directed to the corresponding author.

## Supplementary Materials

The following supporting information can be downloaded at: https://www.mdpi.com/article/10.3390/jcm13226666/s1, Table S1: Literature search strategy, Table S2: Certainty assessment, Table S3: Risk of Bias Assessment, Table S4: Adverse events (AE) reported in the included trials. Adverse events were not serious and did not require discontinuation of the drug.
