# Supplementary material for: Comparative Efficacy of Different Pharmacological Treatments for Pityriasis Rosea: A Network Meta-Analysis
Source: J Clin Med. 2024 Nov 6;13(22):6666. doi: 10.3390/jcm13226666 (PMC11595004; doi:10.3390/jcm13226666)
Supplement: Supplementary file 1 [file jcm-13-06666-s001.zip › jcm-3241238-supplementary.pdf]

Supplementary Table S1. Literature search strategy

CENTRAL (Cochrane Library) search strategy
#1 pityriasis and (rosea or Gibert or Vidal or circinata or marginata or maculata)
#2 MeSH descriptor: [Pityriasis Rosea] This term only
#3 #1 or #2

MEDLINE (Ovid) search strategy
1. randomized controlled trial. pt.
2. controlled clinical trial.pt.
3. randomized.ab.
4. placebo.ab.
5. clinical trials as topic.sh.
6. randomly.ab.
7. trial.ti.
8. 1 or 2 or 3 or 4 or 5 or 6 or 7
9. exp animals/ not humans.sh.
10. 8 not 9
11. Pityriasis Rosea/
12. pityriasis rosea.mp.
13. (pityriasis and Gibert).mp.
14. (pityriasis and Vidal).mp.
15. pityriasis circinata et marginata.mp.
16. pityriasis maculata et circinata.mp.
17. (pityriasis and circinata).mp.
18. (pityriasis and marginata).mp.
19. (pityriasis and maculata).mp.
20. or/11-19
21. 10 and 20

Embase (Ovid) search strategy
1. pityriasis rosea/
2. pityriasis rosea.mp.
3. (pityriasis and Gibert).mp.
4. (pityriasis and Vidal).mp.
5. pityriasis circinata et marginata.mp.
6. pityriasis maculata et circinata.mp.
7. (pityriasis and circinata).mp.
8. (pityriasis and marginata).mp.
9. (pityriasis and maculata).mp.
10. or/1-9
11. crossover procedure.sh.
12. double-blind procedure.sh.
13. single-blind procedure.sh.
14. (crossover or cross-over).tw.
15. placebo.tw.
16. (double and blind).tw.
17. allocate.tw.
18. trial.ti.
19. randomized controlled trial.sh.
20. random.tw.
21. or/11-20
22. exp animal/ or exp invertebrate/ or animal experiment/ or animal model/ or animal tissue/ or animal cell/ or nonhuman/

23. human/ or normal human/

24. 22 and 23
25. 22 not 24
26. 21 not 25
27. 10 and 26

Search strategy for trials registers

Pityriasis rosea
Pityriasis Vidal
Pityriasis Gibert
Pityriasis circinata
Pityriasis marginata

Search strategy for adverse effects (PubMed)

(Drug hypersensitivity [mh] OR Drug toxicity [mh] OR Product surveillance, postmarketing [mh] OR safety [mh] OR adverse effects
[Subheading] OR chemically induced [Subheading] OR Adverse [tw] OR side effect* [tw] OR toxicity [tw] OR chemically-induced [tw] OR
safety [tw]) AND (Pityriasis rosea [mh] OR Pityriasis rosea [tw] OR Pityriasis marginata [tw])

Supplementary Table S2. Certainty assessment.

№ of studies	Study design	Risk of bias	Inconsistency	Indirectness	Imprecision	Publication bias	Certainty
**Itch resolution**							
9	randomized trials	low	not serious	Serious[a]	serious[b]	Not serious	⊕⊕◯◯◯ Low
**Improvement of the skin eruption**							
12	randomized trials	low	not serious	Serious[a]	serious[b]	Not serious	⊕⊕◯◯◯ Low

a. Heterogeneous definition of the outcome across the included RCTs.

b. Wide confidence intervals crossing unity or failure to reach the optimal information size.

Supplementary Table S3. Risk of Bias Assessment.

	Randomization process	Deviations from intended interventions	Missing outcome data	Measurement of the outcome	Selection of the reported result
Lazaro-Medina 1996	L	L	L	L	L
Villarama 2002	L	L	L	L	L
Akhyani 2003	L	L	L	L	L
Amer 2006	L	L	L	L	L
Rassai 2011	L	L	L	L	L
Ahmed 2014	L	L	L	L	L
Ganguly 2014	L	L	L	L	L
Pandhi 2014	L	L	L	L	L
Das 2015	L	L	L	L	L
Singh 2016	L	L	L	L	L
Sonthalia 2018	L	L	L	L	L

L, low; H, high;

Supplementary Table S4. Adverse events (AE) reported in the included trials. Adverse events were not serious and did not require discontinuation of the drug.

Study	Treatment 1	Treatment 2
Lazaro-Medina 1996	None	None
Villarama, 2002	Gastrointestinal AE	Gastrointestinal AE
Akhyani, 2003	Not evaluated	Not evaluated
Amer 2006	Stomach ache in 2 patients, Diarrhoea in 2 patients	No AE
Ehsani, 2010	Not evaluated	Not evaluated
Rassai, 2011	Not evaluated	Not evaluated
Ahmed, 2014	Not evaluated	Not evaluated
Ganguly, 2014	Not evaluated	Not evaluated
Pandhi, 2014	Stomach ache in 3 patients	No AE
Das, 2015	Headache in 3 patients, increased sleep in 2, nausea and vomiting in 2, dysgeusia in 1	Increased sleep in 1 patient
Singh, 2016	No AE	Abdominal pain and diarrhea in 1 patient
Sonthalia, 2018	Mild gastric hyperacidity in 2 patients; transient anxiety and palpitations in one patient	Belching in one patient; stye in one patient
